# Bacterial Pathogens and Symbionts Harboured by *Ixodes ricinus* Ticks Parasitising Red Squirrels in the United Kingdom

**DOI:** 10.3390/pathogens10040458

**Published:** 2021-04-11

**Authors:** Lisa Luu, Ana M. Palomar, Gemma Farrington, Anna-Katarina Schilling, Shonnette Premchand-Branker, John McGarry, Benjamin L. Makepeace, Anna Meredith, Lesley Bell-Sakyi

**Affiliations:** 1 Institute of Infection, Veterinary and Ecological Sciences, University of Liverpool, Liverpool L3 5RF, UK; lisaluu@liverpool.ac.uk (L.L.); gemmafarrington98@gmail.com (G.F.); J.W.Mcgarry@liverpool.ac.uk (J.M.); blm1@liverpool.ac.uk (B.L.M.); 2 Centre of Rickettsiosis and Arthropod-Borne Diseases, Hospital Universitario San Pedro-CIBIR, 26006 Logroño, Spain; ampalomar@riojasalud.es; 3 Royal (Dick) School of Veterinary Studies, University of Edinburgh, Easter Bush, Midlothian EH25 9RG, UK; annakatarinaschilling@gmail.com (A.-K.S.); anna.meredith@unimelb.edu.au (A.M.); 4 Department of Biology, University of York, Wentworth Way, York YO10 5DD, UK; shonnette@hotmail.com; 5 The Pirbright Institute, Ash Road, Pirbright, Surrey GU24 0NF, UK; 6 Melbourne Veterinary School, Faculty of Veterinary and Agricultural Sciences, University of Melbourne, Parkville, Victoria 3010, Australia

**Keywords:** red squirrel, tick, *Ixodes ricinus*, bacteria, *Borrelia*, *Anaplasma phagocytophilum*, *Ehrlichia*, *Spiroplasma*, *Ixodiphagus*, *Wolbachia*

## Abstract

Red squirrels (*Sciurus vulgaris*) are native to most of Eurasia; in much of the United Kingdom, they have been supplanted by the non-native grey squirrel, and are considered an endangered species. Very little is known about the range of tick-borne pathogens to which UK red squirrels are exposed. As part of trap-and-release surveys examining prevalence of *Mycobacterium* spp. in red squirrel populations on two UK islands, *Ixodes ricinus* ticks were removed from squirrels and PCR screened for *Borrelia* spp., intracellular arthropod-borne bacteria and the parasitic wasp *Ixodiphagus hookeri*. At both sites, the most commonly encountered tick-transmitted bacterium was *Borrelia burgdorferi* sensu lato (overall minimum prevalence 12.7%), followed by *Anaplasma phagocytophilum* (overall minimum prevalence 1.6%). Single ticks infected with *Spiroplasma* were found at both sites, and single ticks infected with *Borrelia miyamotoi* or an *Ehrlichia* sp. at one site. Ticks harbouring *Wolbachia* (overall minimum prevalence 15.2%) were all positive for *I. hookeri*. Our study shows that UK red squirrels are potentially exposed to a variety of bacterial pathogens via feeding ticks. The effects on the health and survival of this already vulnerable wildlife species are unknown, and further studies are needed to evaluate the threat posed to red squirrels by *Borrelia* and other tick-borne pathogens.

## 1. Introduction

The Eurasian red squirrel (*Sciurus vulgaris*) is native to large areas of temperate Eurasia, from Ireland and Spain in the west to Japan and the Kamchatka Peninsula in the east [1]; in much of the United Kingdom, it has been supplanted by the non-native eastern grey squirrel (*Sciurus carolinensis*), and is now considered an endangered species. UK red squirrel distribution is currently fragmented, restricted to areas of Scotland and northern England, with isolated populations on a few islands off Wales and the south coast of England [2]. The threat to red squirrels from the squirrel-pox virus, which also infects but is non-pathogenic in grey squirrels, is well documented [3]. Recently, an additional threat to red squirrel health was recognised; infection with *Mycobacterium leprae* and *Mycobacterium lepromatosis*, the causative agents of leprosy, has been reported from several locations in the British Isles [4,5]. In Scotland and continental Europe, red squirrels have been reported as hosts for *Ixodes* spp. ticks and the tick-associated pathogens *Borrelia burgdorferi* sensu lato, *Borrelia miyamotoi*, *Anaplasma phagocytophilum* and *Bartonella washoensis* [6,7,8,9,10,11,12,13,14], although the effect of these microorganisms on squirrel health is unclear. Nothing is known about the range of tick-borne pathogens, and other microorganisms with pathogenic potential such as *Spiroplasma* [15,16], to which UK red squirrels are exposed.

The present study was carried out as part of a larger survey of *Mycobacterium* spp. infection in red squirrels on two UK islands, Brownsea Island off the south coast of England, and the Isle of Arran off the west coast of Scotland [5,17]. Ticks removed from squirrels examined during trap-and-release surveys carried out in 2016–2018 were identified and screened by PCR for presence of a range of pathogenic and symbiotic tick-borne bacteria, as well as the parasitic wasp *Ixodiphagus hookeri*, recently proposed to interfere with pathogen transmission [18], and its *Wolbachia* symbiont.

## 2. Results

### 2.1. Ticks

**Figure 1:**
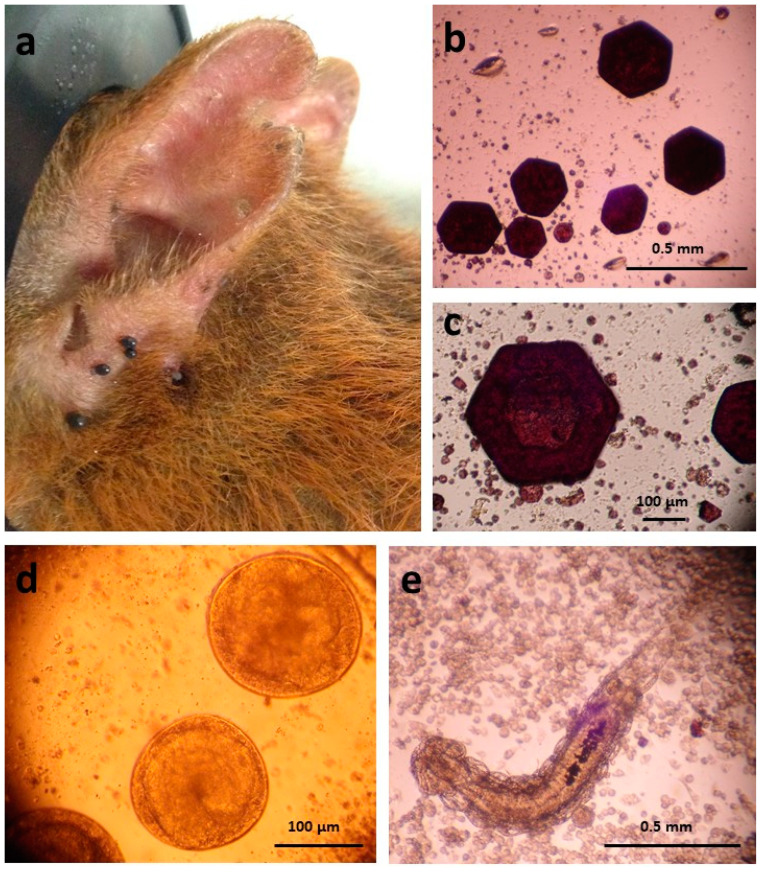
(**a**) Ticks feeding on the base of a red squirrel’s ear, Brownsea Island, Spring 2017; (**b**,**c**) large, hexagonal crystals released from the midgut of a partially-engorged *Ixodes ricinus* nymph dissected in Hanks balanced salt solution (observed live by inverted microscope); (**d**,**e**) *Ixodiphagus hookeri* eggs and larva, respectively, removed from the body cavity of the *I. ricinus* nymph during dissection (observed live by inverted microscope).

In total, red squirrels were examined in 126 trapping events on Brownsea Island (Autumn 2016–Autumn 2018) and 62 trapping events on the Isle of Arran (Spring 2017–Autumn 2018) (Table 1). Not all trapped squirrels carried ticks, and in some cases, ticks were too small to be removed without damaging them. Overall, 88.9% of Brownsea Island squirrels were infested with ticks (Figure 1a) at the time of sampling (range 60–100%), while overall infestation rates on Arran were lower, at 53.2% (range 7.1–100%) (Table 1). Morphological and molecular screening was carried out on 398 ticks removed from 93 squirrels trapped on Brownsea Island, and 39 ticks removed from 24 squirrels on Arran individually (n = 145) or in pools (n = 60) of 2–16 individuals (Table 1). The majority of ticks sampled were nymphs; when larvae were sampled, they were pooled with nymphs from the same host.

All ticks were identified as belonging to the genus *Ixodes*, and all individuals subjected to morphological analysis were identified as *Ixodes ricinus*. Sequence analysis of tick 16S rRNA PCR products from a subset of 42 of these ticks confirmed them to be *I. ricinus*. Fourteen variants were found; ten were identical to sequences from *I. ricinus* previously deposited in GenBank (Table S1), and four showed only a single nucleotide substitution compared to published *I. ricinus* sequences. Amongst the former variants, the nucleotide sequence amplified from a single nymph collected on Brownsea Island in Autumn 2016 also shared a high identity (99.5%) with nucleotide sequences from *Ixodes inopinatus*. (Supplementary Table S1).

### 2.2. Tick-Borne Bacteria

All DNA samples extracted from pooled ticks, and from all but two of the single ticks, gave positive results for the PCR amplifying a fragment of the tick 16S rRNA gene. These positive samples were screened for presence of tick-borne bacterial genera known to be harboured by *I. ricinus* (*Anaplasma*, *Borrelia*, *Ehrlichia*, *Neoehrlichia, Rickettsia* and *Spiroplasma*) using family- or genus-specific PCR assays. While all samples were tested for the family Anaplasmataceae (encompassing *Anaplasma*, *Ehrlichia* and *Neoehrlichia*) and the genera *Borrelia* and *Spiroplasma*, only a subset of 52 samples from Brownsea Island (27 pools and 25 single ticks, corresponding to 36 larvae and 93 nymphs) were tested with primers specific for relapsing fever group *Borrelia* spp. (Table 2). Moreover, 98 of the ticks (9 pools and 39 single ticks) collected on Brownsea Island in Autumn 2017 and Autumn 2018 were not screened for *Rickettsia* spp.

The most frequently-detected bacterium in all years at both sampling sites was *B. burgdorferi* sensu lato, with an overall minimum prevalence of 12.7% (Table 2). On Brownsea Island, with an overall minimum prevalence of 11.6%, *Borrelia afzelii* showed the highest prevalence (minimum 8.3%), followed by *Borrelia garinii* (minimum 2.3%) and *Borrelia valaisiana* (minimum 0.8%). A single tick pool, comprising one larva and two nymphs, was positive for both *B. afzelii* and *B. garinii*. Amongst the ticks tested for relapsing fever *Borrelia* spp., a single tick pool was positive for *B. miyamotoi* giving a minimum prevalence of 0.7%. On Arran, with an overall minimum prevalence of 23.1%, *B. burgdorferi* sensu stricto and *B. garinii* were both detected with minimum prevalences of 7.7%, followed by *B. valaisiana* (5.1%) and *B. afzelii* (2.6%).

Analysis of the *Borrelia* spp. sequences revealed the presence of five *B. garinii*, three *B. afzelii*, two *B. valaisiana*, two *B. burgdorferi* s.s. and one *B. miyamotoi* variants (Table S1). Regarding *B. garinii*, ten out of the 12 amplicons were identical to published *B. garinii* strains. In the remaining two amplicons, from pooled ticks, nucleotide sequences with ambiguous bases were obtained. One more amplicon from a pool showed ambiguous bases and a combination of the most prevalent *B. garinii* and *B. afzelii* variants were identified (Table S1). In addition to this sample, infection with *B. afzelii* was detected in 34 samples of which 30 nucleotide sequences were identical to published sequences. The remaining four amplicons were not identical to any published sequences, but high identities were shared with sequences available from GenBank (Table S1). Five and three amplicons showed 100% identity with, respectively, published *B. valaisiana* and *B. burgdorferi* s.s. sequences (Table S1). Using the relapsing fever *Borrelia*-PCR assay that amplifies the *glpQ* gene, one nucleotide sequence identical to published *B. miyamotoi* sequences was obtained (Table S1).

The PCR assay targeting the Anaplasmataceae *groELS* gene amplified products from 10 and three samples from Brownsea Island and Arran, respectively, although nucleotide sequences were only recovered from seven amplicons in total (Table 3). *A. phagocytophilum* was identified at both sites with overall minimum prevalence on Brownsea Island of 1.3% and on Arran of 5.1% (Table 3). Analysis of the *groELS* sequences revealed the presence of a variant identical to strains pathogenic to humans, such as *A. phagocytophilum* HZ and HZ2, isolated from patients in the United States (CP000235 and CP006616) (Table S1). This variant was amplified from a pool of ticks from Brownsea Island collected in 2016. *A. phagocytophylum* was also detected in six more samples, with five genetic variants all belonging to ecotype I, associated with *I. ricinus* as a vector and a wide range of mammalian hosts [19]. Specifically, one sequence was identical to that of *A. phagocytophylum* from a sheep in Norway (AF548385), and five amplicons were closely related (99.8–99.9% identity) to published *A. phagocytophilum* sequences (HM057224, AY281828, AF478553 and AY281831) (Table S1), all belonging to ecotype I [19].

An *Ehrlichia* sp. was detected in a single tick from Brownsea Island in Spring 2017 (Table 3). The analysis of the *groELS* gene fragment showed the highest identity (99.5%, 100% query cover) with an unclassified *Ehrlichia* sp. detected in *Hyalomma asiaticum* from China (JX402611). With a query cover of 94%, this sample reached identities of 99.4% with *Ehrlichia minasensis* (JX629806) and 97.5% with *Ehrlichia canis* (CP025749). The *Ehrlichia* was further characterised by amplification of a *gltA* gene fragment, which showed homology (100% identity) with sequences of *E. canis* (CP000107) (Table S1).

A *Spiroplasma* sp. was detected in one tick pool from each site (Table 3). The 16S rRNA sequences obtained from both samples showed 100% identity with *Spiroplasma* sp. strain Bratislava1 isolated from Slovakian *I. ricinus* (KP967685), but also with other *Spiroplasma* strains isolated from Japanese *Ixodes persulcatus* and *Ixodes monospinosus* (LC388762 and LC388760) (Table S1). An *rpoB* amplicon was only obtained from the tick pool from Arran and was identical to the sequence of *Spiroplasma* sp. strain Bratislava1 (KP967687). Co-infections with any of the tick-borne bacteria (i.e., excluding *Wolbachia*) were not detected in single ticks, and none of the tested samples were positive by PCR for any *Neoehrlichia* or *Rickettsia* spp.

### 2.3. Ixodiphagus and Wolbachia

Thirteen nymphs removed from a subset of nine Brownsea Island squirrels sampled in Spring 2017 were dissected live. Interestingly, the midgut contents released during dissection of the live ticks contained large, hexagonal, dark red crystals (up to ~300 µm across) that were presumably products of partial digestion of the squirrel blood (Figure 1b,c). Over half of the dissected nymphs contained between one and nine *Ixodiphagus* sp. eggs or larvae (Figure 1d,e). Amplification of a fragment of the cytochrome oxidase I (COI) gene from DNA extracted from the *Ixodiphagus* eggs (n = 10) and larvae (n = 14) confirmed the species as *I. hookeri* (100% identity to published sequence JQ315225), and presence of *Wolbachia* therein was confirmed by PCR amplification of a fragment of the *Wolbachia* 16S rRNA gene.

Amongst Brownsea Island ticks, 20/56 (35.7%) pools and 52/118 (44.1%) individual ticks were positive by PCR for presence of *Ixodiphagus*. When the same samples were screened for *Wolbachia* DNA, all of the *Ixodiphagus*-positive tick pools and 46/52 (88.5%) *Ixodiphagus*-positive individual ticks were positive for *Wolbachia* (Table 3). *Wolbachia* DNA was not detected in any tick pools or individual ticks that were negative for *Ixodiphagus*; however, six individual ticks were positive for *Ixodiphagus* but negative for *Wolbachia*. Neither organism was detected in ticks collected on the Isle of Arran.

Seventeen *Ixodiphagus* COI amplicons were randomly selected, mainly from single ticks, and sequenced. The nucleotide sequences were identical to the single publicly available comparable full-length sequence of *I. hookeri* (JQ315225) (Table S1). Analysis of 51 of the *Wolbachia* 16S rRNA sequences obtained from tick samples revealed the presence of at least three variants, two of which were identical to the only published sequence of a *Wolbachia* from *I. hookeri* (KU255240) but with query cover of only 59.3% (Table S1). When considering 100% query cover, the most prevalent variant was identical to a *Wolbachia* sp. detected in the leaf beetle *Diabrotica lemniscate* (AY007547). The other two variants were not identical to published sequences but one of them was closely related to the *D. lemniscate Wolbachia*, with only one base substitution. The third variant showed the highest identity (99.9%) with a *Wolbachia* sp. detected in a *Notoncus* sp. ant (GQ275137) (Table S1). We also obtained sequences with ambiguous bases that were closely related to the aforementioned variants, amongst others (Table S1). These nucleotide sequences were amplified both from tick pools and from single ticks that could have been parasitized by multiple eggs/larvae deposited by more than one individual wasp, and could therefore have harboured more than one *Wolbachia* variant.

**Figure 2:**
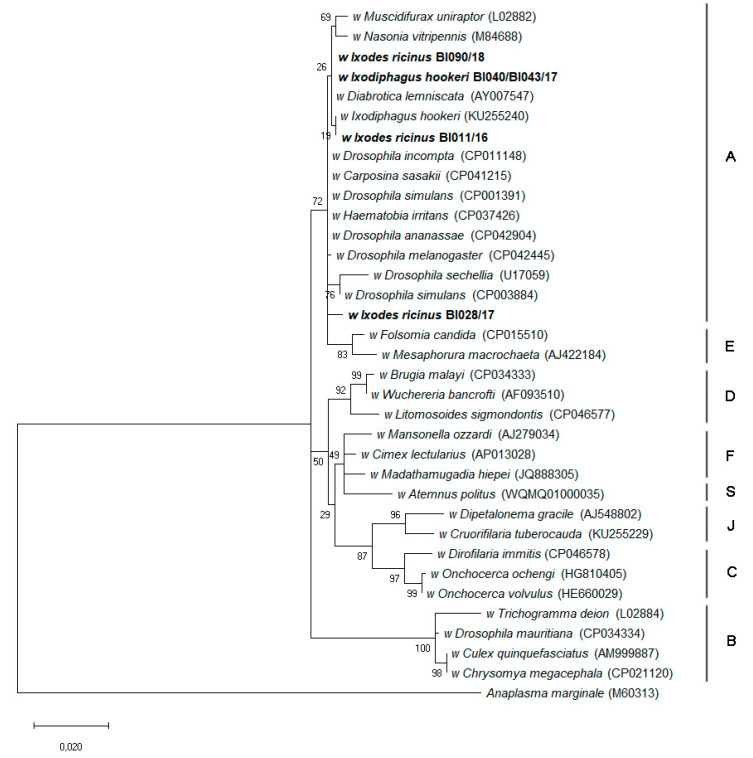
Phylogenetic analysis of *Wolbachia* 16S rRNA gene sequences (in bold) obtained in this study from *Ixodiphagus hookeri* eggs and *Ixodes ricinus* ticks, and published sequences from *I. hookeri*, other insects and nematodes known to harbour *Wolbachia*. *Anaplasma marginale* was used as an outgroup, and *Wolbachia* supergroups are indicated by upper-case letters to the right of the tree. The maximum likelihood tree was based on 35 partial 16S rRNA gene sequences with a total of 972 positions in the final dataset. The phylogeny was inferred with the Tamura-Nei model, a discrete Gamma-distribution and a proportion of invariable sites (TN93+G+I). The tree is drawn to scale, with branch lengths measured in the number of substitutions per site. Numbers represent bootstrap support generated from 500 iterations. The GenBank accession numbers of the sequences used in these analyses are shown in brackets.

The *Wolbachia* sequence obtained from the sample of *I. hookeri* eggs was identical to the variant obtained from tick samples with 100% identity to the *D. lemniscate Wolbachia* (AY007547) (Table S1). However, the *Wolbachia* sequence obtained from the *I. hookeri* larvae was almost identical to the sequence from the eggs, but contained a single ambiguous base at position 1172 of the published sequence (AY007547) (Table S1). At this position the *D. lemniscate Wolbachia* has a C; our sequence has a Y (i.e., C or T) at this position, suggesting the presence of a mixed population. The sequence with T at this position did not match with any published *Wolbachia* sequences. This sequence was identical to one of the variants with ambiguous bases mentioned above, amplified from ten tick samples (5 pools, 5 single ticks). Phylogenetic analysis based on a 970 bp fragment of the *Wolbachia* 16S rRNA gene placed all the Brownsea Island sequences in Supergroup A, together with the only comparable published sequence from an *I. hookeri* wasp (Figure 2).

### 2.4. Sequences Submitted to a Public Database

Fourteen nucleotide sequences amplified in this study that were not identical to already-published sequences, and the *Wolbachia* sequence obtained from *I. hookeri* eggs, were deposited in the GenBank database under the accession numbers shown in the third column and footnote of Table S1 (MW727241–MW727243, MW727259–MW727263 and MW732490–MW732496).

## 3. Discussion

Red squirrels trapped at the two UK sampling sites were frequently infested with *I. ricinus* ticks, generally confirming observations on this host species in Scotland and continental Europe. In a study of UK red squirrels examined postmortem, 25% of squirrels from unspecified locations in Scotland were carrying ticks identified as *I. ricinus*, while no ticks were found on squirrels from the Isle of Wight, an island less than 30 km east of Brownsea Island [9]. An earlier study found that less than 10% of Scottish red squirrels, also examined postmortem, were infested with ticks of unspecified species [20]. In Switzerland, 5/6 road-kill squirrels collected in the Neuchatel area were carrying between 12 and 747 ticks, predominantly larvae and nymphs [6]. Amongst 237 red squirrels, mostly roadkill, examined from different areas of France, 88 adults (37.1%) were carrying between 1 and 415 immature ticks, almost all of which were *I. ricinus* [10]. Similarly, in a study of road-kill red squirrels from France and Italy, 34.0% of 311 French squirrels carried *I. ricinus* ticks, whereas only 6.5% of Italian squirrels were tick-infested and in the latter case the tick species was *Ixodes acuminatus* [8]. In Lithuania, 9/39 road-kill red squirrels were carrying *I. ricinus* ticks [13].

All the ticks analysed in this study were identified as *I. ricinus*, although one of the genetically identified variants was identical to several published *I. ricinus* sequences and closely related, but not identical, to an *I. inopinatus* haplotype. The known low genetic divergence between these two species, based on a small fragment of the 16S rRNA gene, and the increase in intraspecific heterogeneity according to sequences published in GenBank, make an accurate genetic identification of this variant difficult. *I. inopinatus* was first described in the Mediterranean region [21], but within a few years it was also reported from Central and Northern Europe [22,23], but not yet from the UK. *I. ricinus* and *I. inopinatus* are sympatric species in some areas, and differences in ecological niche, host preference or microbiota between the two species have not been demonstrated [23,24].

Ticks infected with members of the *B. burgdorferi* s.l. genospecies were found in all years, at both sites, with a minimum overall prevalence of 12.6%. This compares with overall *Borrelia* prevalences of *I. ricinus* of 1.7% (range 0–6.0%) detected in questing nymphs sampled at multiple sites across mainland Scotland and the Isle of Mull [25], 6.4% and 0.7% in questing nymphs at sites of, respectively, high and low Lyme disease incidence in the Outer Hebrides [26], 3.2% (range 0–5.6%) in questing nymphs and adults at four sites in England [27], 19.0% (range 0–24.5%) in questing nymphs collected over three years at sites in a single area of southern England [28] and 3.8% (range 0–24.0%) in questing ticks collected over six years at 20 sites in recreational areas of England and Wales [29]. In the two last-mentioned studies, significant variation was seen in tick infection rates between years [28,29]; on Brownsea Island, where ticks were sampled in both Spring and Autumn, the minimum overall prevalence of *Borrelia* infection was 9.9% in 2017 compared with 21.3% in 2018. As the ticks analysed in the present study had all fed to some extent on their red squirrel hosts, and no questing ticks were collected from the same sites for comparison, it was not possible to estimate whether *Borrelia* prevalence was due entirely to prior infection in the ticks or was enhanced by presence of spirochaetes in the blood meal.

In Brownsea Island ticks, *B. afzelii* was the most frequently encountered species, comprising two-thirds of all positive samples, followed by *B. garinii* and *B. valaisiana*; all three species were detected in ticks from the Isle of Arran, where *B. burgdorferi* s.s. was also found, but where numbers were too low to draw conclusions on relative prevalence between species. These results agree with previous reports of *B. afzelii*, *B. garinii* and *B. valaisiana*, but not *B. burgdorferi* s.s. in questing ticks in southern England [27,28,29] and all four species in questing ticks in Scotland [25]. However, in both studies on ticks from southern England, *B. garinii* was the predominant species, whereas *B. afzelii* predominated in the Brownsea Island ticks.

The host upon which ticks last fed may influence the distribution of *Borrelia* genospecies in *I. ricinus*; in the present study, all sampled ticks had last fed on the same host species (red squirrels) although in the case of nymphs, the larval hosts were unknown and could have been red squirrels or other small vertebrates present in the same area. Red squirrels are not present at the locations sampled in the two southern England studies [27,28], whereas they could have hosted at least some of the ticks sampled in one of the Scottish studies [25]. Spirochaetes were isolated from 90/227 ticks removed from red squirrels in Switzerland; of these, 43 were *B. afzelii*, 33 were *B. burgdorferi* s.s., four were *B. garinii* and ten were a mixture of *B. afzelii* and *B. burgdorferi* s.s. [6]. Previous studies have reported presence of DNA from *B. burgdorferi* s.s., *B. afzelii* and *B. garinii* in red squirrels in Norway, France and Czech Republic, and from *B. afzelii* in The Netherlands [7,10,11,12,14]. *B. valaisiana* has not been reported to infect red squirrels.

*B. miyamotoi* has been previously reported to occur at very low prevalence in questing *I. ricinus* collected at several sites in southern and eastern England [27,28,29,30] and in 1/ 153 nymphs removed from humans participating in a long-distance running event in the Scottish Highlands [31]. The very low prevalence of *B. miyamotoi* found in Brownsea Island ticks is in agreement with these reports. In contrast, 6.7% (3/45) and 13.6% (3/22) of red squirrels tested in The Netherlands and Czech Republic, respectively, were carrying *B. miyamotoi* DNA [11,14]. Several human cases of *B. miyamotoi* infection have been reported in mainland Europe, but not as yet in the UK [32].

Much less attention has been focused on screening for members of the Rickettsiales in red squirrels or the ticks infesting them. None of the studies cited above tested ticks removed from red squirrels for presence of *Anaplasma* spp., *Ehrlichia* spp., *Rickettsia* spp. or *Neoehrlichia* spp., although *A. phagocytophilum*, but not *Candidatus* Neoehrlichia mikurensis, was detected in spleen samples from 8.9% (4/45) red squirrels in The Netherlands [11]. *A. phagocytophilum*, the causative agent of tick-borne fever in domestic ruminants and human granulocytic anaplasmosis [33] has been detected at low prevalence in *I. ricinus* ticks in many parts of the UK [34,35,36]. In the present study, an overall minimum *A. phagocytophilum* prevalence of 1.6% was found in *I. ricinus* removed from red squirrels, with positive ticks at both sites. All the detected variants belong to ecotype I, the most widely-distributed and abundant ecotype in Europe, with a broad host range that includes humans [19] and red squirrels [11]. A variant with a *groELS* sequence identical to *A. phagocytophilum* strains HZ [37] and HZ2, involved in human cases in the US, was detected in a tick pool from Brownsea Island.

A single Brownsea Island tick was positive for an *Ehrlichia* sp. with close identity to *E. minasensis* and *E. canis*, pathogens of, respectively, cattle and dogs with worldwide distribution; no previous reports could be found of UK *I. ricinus* ticks infected with any bacteria currently classified in the genus *Ehrlichia*. DNA from *Rickettsia* spp. was not detected in the present study, although spotted fever group *Rickettsia* were detected in 6.5% of *I. ricinus* ticks collected from vegetation and hosts in northern Scotland and southern England [38]. On the other hand, the absence of *Ca*. N. mikurensis DNA in ticks from both sampling sites in the present study agreed with a previous report in which this pathogen was not detected in any of 401 ticks collected in the UK [39]. Similarly, no reports could be found of UK ticks carrying any *Spiroplasma* spp., although this bacterium has been detected in, and isolated from, *I. ricinus* in continental Europe [16]. The present study therefore constitutes the first molecular detection of bacteria of two genera, *Ehrlichia* and *Spiroplasma*, in UK *I. ricinus* ticks.

The genus *Wolbachia*, predominantly carried by insects, but also found in other arthropods and nematodes, comprises a wide range of “supergroups” (high-level clades) with a variety of parasitic, mutualistic or ambiguous relationships with their invertebrate hosts. A number of studies have reported presence of *Wolbachia* DNA in *I. ricinus* ticks from Italy, Germany, France, The Netherlands and Morocco [40,41,42,43,44,45,46]. However, as ticks can themselves be parasitised by insects, namely the wasp *I. hookeri*, as well as by nematodes and mites, it is unclear whether or not *Wolbachia* ever naturally infects tick tissues. While a recent study demonstrated that several strains of *Wolbachia* can infect and grow in *Ixodes* spp. tick cells in vitro [47], this does not prove that ticks themselves carry the bacterium in vivo. Most of the above-mentioned studies on whole ticks did not include a screen for insect or helminth DNA; of those that did, both found a strong correlation between presence of *Wolbachia* and presence of the wasp *I. hookeri* [44,45]. In the present study, the first report of *Ixodiphagus* in the UK since 1943 [48], all tick pools and single ticks that were PCR positive for *Wolbachia* were also PCR positive for *Ixodiphagus*, providing support for the view that *Wolbachia* DNA detected in field ticks results from parasites present in the body of the tick. While a *Wolbachia* infection rate of almost 100% was found in adult *I. hookeri* that emerged from ticks collected from vegetation or roe deer in France, with only one out of 121 wasps (0.8%) negative for the symbiont [45], in the present study, 6/53 individual ticks (11.3%) were positive for *I. hookeri* but negative for *Wolbachia*. This finding suggests that the *Wolbachia* infection rate in *I. hookeri* from the sampled UK environments may be slightly lower than in France, or that a higher *Wolbachia* detection rate can be achieved by sampling adult wasps, rather than immature stages inside ticks.

Although co-infections with multiple pathogenic bacteria have been frequently reported in surveys of questing or host-associated *I. ricinus* [6,7,31,49], and are considered the norm rather than the exception in continental Europe [50], no pathogen co-infections were detected in single ticks collected at either site in the present study. This could be due to the small sample size, to sampling of ticks being restricted to those infesting red squirrels or, in the case of Brownsea Island, to the particular location of the study site on a small island where the diversity of larval hosts and therefore opportunities for picking up a diverse range of pathogens may be limited.

On the other hand, amongst the single ticks on Brownsea Island that were positive for *Borrelia* spp., the incidence of *Borrelia* DNA was over twice as high, at 21.3% in ticks that were also positive for *Ixodiphagus*/*Wolbachia* DNA (10/47), than the 10.8% infection rate in ticks that were negative for *Ixodiphagus*/*Wolbachia* DNA (8/74). As there is no evidence that *Wolbachia* naturally infects ticks, but is rather present within parasitic insects or nematodes infesting ticks [45,47], any effect on co-infecting pathogenic bacteria is less likely to result from the *Wolbachia* than directly from the parasite competing for nutrients or interfering with innate immune responses of the tick host. Infestation of *I. ricinus* nymphs with *I. hookeri* in The Netherlands was found to be positively correlated with presence of *A. phagocytophilum* and negatively correlated with presence of *B. afzelii* and *Ca*. N. mikurensis [18]. Further studies are needed to determine the effect of *I. hookeri* infestation, and indeed presence in the tick microbiome of symbionts such as *Rickettsia*, *Wolbachia* [51] and *Spiroplasma*, on pathogen prevalence in different tick populations, and the mechanisms underlying such effects.

An interesting by-product of the dissection of live ticks for collection of *I. hookeri* eggs and larvae was the observation of predominantly hexagonal haemoglobin crystals derived from the host squirrel blood. Pseudo-tetrahedral or pyramidal and flattened pseudo-tetrahedral haemoglobin crystals were described from gut contents of *Ornithodoros moubata* ticks, *Rhodnius prolixus* kissing bugs and *Xenopsylla cheopis* fleas fed on guinea pigs [52,53,54], while rhomboidal and trapezoidal crystals were found in guts of *X. cheopis* fed on rats [53]. The shape of the haemoglobin crystals appears to depend on the host species, rather than that of the feeding arthropod, as reported previously [53,54]; hexagonal crystals of haemoglobin formed from red squirrel blood were reported more than a century ago [55].

In conclusion, the results of the present study show that UK red squirrels are actually and potentially exposed to a variety of known and potential pathogens via the ticks feeding on them. The effects of such exposure on the health and survival of this already vulnerable wildlife species are unknown. Further studies are needed to evaluate the threat posed to red squirrels by tick-borne pathogens such as *Borrelia* spp. and *A. phagocytophilum*.

## 4. Materials and Methods

### 4.1. Tick Sampling

All applicable national, and/or institutional guidelines for the care and use of animals were followed. All procedures performed in this study were in accordance with the ethical standards of the United Kingdom Government Home Office and approved under Home Office Project Licence (PPL) 70/9023, Natural England Licence 2016–24,517-SCI-SCI, and Scottish Natural Heritage Licence 90,896, with ethical approval from the University of Edinburgh. Sampling was carried out at sites on Brownsea Island (50°69′ N, 1°97′ W), Poole Harbour, Dorset, on the south coast of England, in Autumn 2016 and Spring and Autumn 2017 and 2018, and the Isle of Arran (59°59′ N, 5°21′ W), Ayrshire, off the west coast of Scotland, in Spring and Autumn 2017 and 2018. Red squirrels were live-trapped and temporarily anaesthetised as described previously [56]. As part of the clinical assessment, they were examined for presence of ticks. Attached ticks were removed using forceps and placed into microcentrifuge tubes containing 70% ethanol separately for each squirrel. Ticks were collected live, without 70% ethanol, from a subset of 9 squirrels sampled in March 2017. All ticks were visually identified to genus level. Subsets of 16 nymphs from Brownsea Island and three nymphs from the Isle of Arran were identified morphologically to species level, using a published key [57]. Genetic identification of 42 single ticks (36 from Brownsea Island and 6 from Arran) was carried out by analysis of the tick 16S rRNA gene as described below (Section 4.3). Numbers of pooled and individually processed ticks from each sampled squirrel are summarised in Table 1.

### 4.2. Tick Dissection

Live, partially-engorged nymphs removed from each of the subset of 9 squirrels sampled in March 2017 were surface-sterilised by immersion for 5 min in 0.1% benzalkonium chloride, 1 min in 70% ethanol and a rinse in sterile deionised water, air-dried, and partially embedded dorsal side uppermost in sterile wax as described previously [16]. The dorsal integument was removed under Hanks balanced salt solution (HBSS) and the entire body contents were transferred to a drop of HBSS. Approximately half of the body contents from each tick were frozen at −20 °C before being processed separately for DNA extraction as described below. Eggs and larvae of *Ixodiphagus* present inside the tick body cavities were collected separately into HBSS and then 70% ethanol and frozen at −20 °C for DNA extraction.

### 4.3. DNA Extraction and PCR Analysis

Ticks in 70% ethanol were processed individually (large nymphs) or in pools of 2–16 ticks (small nymphs/larvae) per pool. Ticks were rinsed with sterile water and chopped into several pieces using a sterile scalpel blade. The DNA from each tick or tick pool was extracted using a DNeasy Blood & Tissue kit (Qiagen, Hilden, Germany), following the manufacturer’s recommendations with an overnight lysis and a 10 min incubation at 70 °C after the addition of the AL buffer. DNA was measured by fluorometric quantification (Qubit, Thermo-Fisher, Loughborough, UK). DNA was extracted from the internal organs and the *Ixodiphagus* samples from dissected live ticks following the same extraction protocol.

Adequate DNA extraction and the control of inhibitors of the PCR technique in the tick samples were checked using a PCR assay that amplified a 456 bp fragment of the tick 16S rRNA gene [58]. Samples that gave positive results were then analysed for presence of genes of *Borrelia* (*flaB* and *glpQ*) [59,60,61,62], *Anaplasmataceae* (*groELS*) [63], *Ehrlichia* (*gltA* for *groELS*-positive samples) [64,65], *Rickettsia* (*ompB*) [66], *Spiroplasma* (*rpoB*, and *16S rRNA* for *rpoB*-positive samples) [67,68] and *Wolbachia* (16S rRNA) [69,70] using genus- or family-specific PCR assays targeting the respective indicated genes as described by the respective authors. DNA samples were also assayed for the presence of *Ixodiphagus* spp. using a PCR assay amplifying a 268 bp fragment of the COI gene [45]. These assays were carried out as described by the respective authors using BioMix™ Red (Bioline Reagents Ltd., London, UK), following the manufacturer’s recommendations for a final volume of 30 µL. A total of 40–50 ng of DNA was added per reaction. Two negative controls (one for extraction and one with water instead of DNA) were included in the PCR assays along with appropriate positive control DNA as follows: *B. burgdorferi* s.s. strain KS20 (kindly provided by Prof. Brian Stevenson, University of Kentucky, Lexington, KT, USA) or *Borrelia spielmanii* (kindly provided by Dr Volker Fingerle, German National Reference Centre for *Borrelia*, Oberschleißheim, Bavaria, Germany); *A. phagocytophilum* strain Webster (kindly provided by Prof Didier Raoult, Unité de Recherche sur les Maladies Infectieuses et Tropicales Emergentes, Marseille, France and Prof Stephen Dumler, The Johns Hopkins Hospital, Baltimore, MR, USA) or strain Feral Goat grown in tick cells [71], *Ehrlichia ruminantium* strain Ball3 grown in tick cells [72], *Rickettsia amblyommatis* [73]; *Spiroplasma* sp. strain Bratislava1 [74] or strain DMAR11 [16], *Wolbachia* strain wAlbB grown in mosquito or tick cells [47], and extract of eggs and larvae of *I. hookeri* (obtained in the present study).

### 4.4. Sequence and Phylogenetic Analyses

PCR products were visualised by 1% agarose gel electrophoresis. Samples showing products of the expected size were cleaned using a Monarch^®^ DNA Gel Extraction kit or Monarch^®^ PCR and DNA Cleanup kit (New England Biolabs, Inc.) and subjected to Sanger sequencing. PCR products were sequenced in both senses and the nucleotide sequences were analysed and compared to those available in the NCBI database using BLAST (http://blast.ncbi.nlm.nih.gov/Blast.cgi accessed on 30 November 2020). Sequences were aligned using the European Bioinformatics Institute multisequence software Clustal Omega (https://www.ebi.ac.uk/Tools/msa/clustalo/ accessed on 30 November 2020) for multiple sequence alignment. The resultant sequences that differed from all other available published sequences were submitted to GenBank using the submission portal (https://submit.ncbi.nlm.nih.gov/subs/genbank accessed on 30 November 2020) and BankIt (https://www.ncbi.nlm.nih.gov/WebSub/ accessed on 30 November 2020). Phylogenetic analysis was conducted with MEGA version X (http://www.megasoftware.net accessed on 30 November 2020) using the maximum likelihood method including all sites. The nucleotide substitution model (TN93+G+I) was selected according to the Akaike information criterion [75] implemented in Mega X. Confidence values for individual branches of the resulting trees were determined by bootstrap analysis with 500 replicates. The published sequences used in the analysis are shown in the phylogenetic tree.

## Figures and Tables

**Table 1:** Numbers of squirrel examinations on Brownsea Island and Arran between Autumn 2016 and Autumn 2018, tick infestation rates and numbers of larval and nymphal ticks screened as pools or single ticks.

Site/Year	Squirrels Trapped	Squirrels with Ticks (%)	Squirrels Whose Ticks Were Screened	Ticks Screened (Larvae, Nymphs)	Pools	Single Ticks
Brownsea/Autumn 2016	26	25 (96.2%)	19	81 (33, 48)	18	1
Brownsea/Spring 2017	26	24 (92.3%)	11	13 (0, 13)	0	13
Brownsea/Autumn 2017	20	20 (100%)	20	211 (0, 211)^1^	29	39
Brownsea/Spring 2018	25	15 (60.0%)	15	43 (3, 40)	9	19
Brownsea/Autumn 2018	29	28 (96.6%)	28	51 (0, 51)	0	51
**Brownsea total**	**126**	**112 (88.9%)**	**93**	**399 (36, 363) ^1^**	**56**	**123**
Arran/Spring 2017	17	4 (23.5%)	0			
Arran/Autumn 2017	6	6 (100%)	6	21 (0, 21)	4	4
Arran/Spring 2018	14	1 (7.1%)	0			
Arran/Autumn 2018	25	22 (88.0%)	18	18 (0, 18)	0	18
**Arran total**	**62**	**33 (53.2%)**	**24**	**39 (0, 39)**	**4**	**22**

^1^ Two individual tick samples did not yield sufficient DNA for molecular analysis.

**Table 2:** Incidence of *Borrelia burgdorferi* s.l. and *Borrelia miyamotoi* DNA in pooled and single *Ixodes ricinus* ticks removed from red squirrels and screened by PCR using genus- or species-specific assays.

Site and Year	No. of Ticks Sampled ^1^	No. *Borrelia* Positive ^1^	*Borrelia afzelii* ^1^	*Borrelia burgdorferi* s.s. ^1^	*Borrelia garinii* ^1^	*Borrelia valaisiana* ^1^	*Borrelia miyamotoi* ^1^
Brownsea Island							
2016 Autumn	81 (18, 1)	5 (5, 0)	5 (5, 0)	0	0	0	1 (1, 0)
2017 Spring	13 (0, 13)	0	0	0	0	0	0
2017 Autumn	209 (29, 37)	22 (19, 3) ^2^	19 (16, 3)	0	2 (2, 0)	1 (1, 0)	ND
2018 Spring	43 (9, 19)	11 (4, 7) ^3^	3 (1, 2)	0	6 (1, 5)	1 (1, 0)	0
2018 Autumn	51 (0, 51)	9 (0, 9) ^4^	6 (0, 6)	0	1 (0, 1)	1 (0, 1)	ND
**Total**	**397 (56, 121)**	**47 (28, 19) ^3^**	**33 (22, 11)**	**0**	**9 (3, 6)**	**3 (2, 1)**	**1**
**Arran**							
2017 Autumn	21 (4, 4)	3 (2, 1)	0	2 (1, 1)	1 (1, 0)	0	ND
2018 Autumn	18 (0, 18)	6 (0, 6)	1 (0, 1)	1 (0, 1)	2 (0, 2)	2 (0, 2)	ND
Total	39 (4, 22)	9 (2, 7)	1 (0, 1)	3 (1, 2)	3 (1, 2)	2 (0, 2)	ND
**Overall minimum prevalence**		**12.8%** **56/436**	**7.8%** **34/436**	**0.7%** **3/436**	**2.8%** **12/436**	**1.1%** **5/436**	**0.7%** **1/136**

^1^ numbers in brackets indicate (number of pools, number of single ticks); ^2^ three additional samples yielded amplification products of the expected size but with insufficient material for sequencing; ^3^ one nucleotide sequence with ambiguous bases that could be a coinfection of *B. garinii* and *B. afzelii* variants was identified and is listed in Supplementary Table S1 as *Borrelia* spp.; ^4^ two additional samples yielded amplification products of the expected size but with insufficient material for sequencing; ND = not done.

**Table 3:** Incidence of *Anaplasma phagocytophilum*, *Ehrlichia* sp., *Spiroplasma* sp. and *Wolbachia* sp. DNA in pooled and single *Ixodes ricinus* ticks removed from red squirrels and screened by PCR using genus-specific assays.

Site and Year	No. of Ticks Sampled ^1^	*Anaplasma phagocytophilum* ^1^	*Ehrlichia* sp. ^1^	*Spiroplasma* sp. ^1^	*Wolbachia* sp. ^1^
Brownsea Island					
2016 Autumn	81 (18, 1)	1 (1, 0)	0	0	10 (9, 1)
2017 Spring	13 (0, 13)	0	1 (0, 1)	0	8 (0, 8)
2017 Autumn	209 (29, 37)	3 (3, 0) ^2^	0	1 (1, 0)	12 (6, 6)
2018 Spring	43 (9, 19)	1 (0, 1)	0	0	13 (5, 8)
2018 Autumn	51 (0, 51)	0 ^3^	0	0 ^3^	24 (0, 24)
**Total**	**397 (56, 121)**	**5 (4, 1)**	**1 (0, 1)**	**1 (1, 0)**	**67 (20, 47)**
**Arran**					
2017 Autumn	21 (4, 4)	2 (2, 0)	0	1 (1, 0)	0
2018 Autumn	18 (0, 18)	0 ^3^	0	0	0
**Total**	**39 (4, 22)**	**2**	**0**	**1 (1, 0)**	**0**
**Overall minimum prevalence**		**1.6%** **(7/436)**	**0.2%** **(1/436)**	**0.5%** **(2/436)**	**15.4%** **(67/436) ^4^**

^1^ numbers in brackets indicate (number of pools, number of single ticks); ^2^ four additional samples yielded amplification products of the expected size but with insufficient material for sequencing; ^3^ one additional sample yielded an amplification product of the expected size but with insufficient material for sequencing; ^4^ 51 out of 67 samples (mainly from single ticks) were sequenced.

## Data Availability

The sequences presented here are available in GenBank and can be identified using the accession numbers presented in Table S1.

## Supplementary Materials

The following is available online at https://www.mdpi.com/article/10.3390/pathogens10040458/s1, Table S1: Highest identities reached between sequences of genes of *Ixodes ricinus* ticks, the parasitic wasp *Ixodiphagus hookeri*, and *Borrelia*, *Anaplasma*, *Ehrlichia*, *Spiroplasma* and *Wolbachia* spp. bacteria detected in the present study and published sequences.
