# Supplementary material for: Bacterial Pathogens and Symbionts Harboured by Ixodes ricinus Ticks Parasitising Red Squirrels in the United Kingdom
Source: Pathogens. 2021 Apr 11;10(4):458. doi: 10.3390/pathogens10040458 (PMC8070177; doi:10.3390/pathogens10040458)
Supplement: Supplementary file 1 [file pathogens-10-00458-s001.pdf]

Table S1. Highest identities reached between sequences of genes of *Ixodes ricinus* ticks, the parasitic wasp *Ixodiphagus hookeri,* and *Borrelia, Anaplasma, Ehrlichia, Spiroplasma* and *Wolbachia* spp. bacteria detected in the present study and published sequences.

Organism	Gene (bp)	No. of sequences obtained (GenBank accession No.)	Origin	Percentage identity with published sequence from species (GenBank accession No.)
Ixodes ricinus	16S rRNA (363-409)	20	BI;IA	100% *I. ricinus* (AF549842-MH645519-KX384805)
		5	BI	100% *I. ricinus* (GU074616-MH645521-KX384811-KF197114)
		3	BI;IA	100% *I. ricinus* (GU074597)
		2	BI	100% *I. ricinus* (KX384806-KF197130-GU074590)
		2	BI	100% *I. ricinus* (HG916806-GU074630)
		2	BI	100% *I. ricinus* (GU074644)
		1	BI	100% *I. ricinus* (GU074601)
		1	BI	100% *I. ricinus* (MK671585)
		1[1] (MW727263)	BI	100% *I. ricinus* (GU074607-GU074618)
		1	BI	100% *I. ricinus* (GU074645-KF197121)
		1 (MW727262)	BI	99.8% *I. ricinus* (MH645519-KX384805)
		1 (MW727261)	BI	99.8% *I. ricinus* (MH645517-KF197116-GU074620)
		1 (MW727260)	BI	99.8% *I. ricinus* (GU074604)
		1 (MW727259)	BI	99.7% *I. ricinus* (HG916806-GU074630)
Ixodiphagus hookeri	COI (215)	17[2]	BI	100% *Ixodiphagus hookeri* (JQ315225)
Borrelia garinii	*flaB* (452)	2	BI;IA	100% *B. garinii* (HM345904-D89899-AY342023-MF150067)
	flaB (334)	4	BI;IA	100% *B. garinii* (CP018744-CP028861)
		2	BI;IA	100% *B. garinii* (AB091807)
		2	BI	100% *B. garinii* (MF150062)
		2[3]	BI	99.8-99.1% *B. garinii* (MF150062-AB091807)
Borrelia afzelii	*flaB* (452)	9	BI	100% *B. afzelii* (CP018262)
		1 (MW732490)	BI	99.8% *B. afzelii* (CP018262)
	flaB (380)	1 (MW732491)	BI	99.7% *B. afzelii* (CP018262)
	flaB (334)	21	BI;IA	100% *B. afzelii* (CP018262)
		2	BI	99.7% *B. afzelii* (CP018262)
Borrelia valaisiana	*flaB* (334)	3	BI;IA	100% *B. valaisina* (CP009117)
		2	BI;IA	100% *B. valaisina* (MF150080)
***Borrelia burgdorferi* s.s.**	*flaB* (334)	2	IA	100% *B. burgdorferi* s.s. (CP001205)
		1	IA	100% *B. burgdorferi* s.s. (CP002312)
***Borrelia* spp.**	*flaB* (334)	1[3]	BI	96.4% *B. garinii* (MF150062)
				95.2% *B. afzelii* (KR782184)
Borrelia miyamotoi	*glpQ* (658)	1	BI	100% *B. miyamotoi* (CP046389-CP044784-MG136725…)
Anaplasma phagocytophilum	*groELS* (958)	1	BI	100% *A. phagocytophilum* (CP000235-CP006616-CP006617-CP006618)
	groELS (1220-1248)	1	IA	100% *A. phagocytophilum* (AF548385)
		1 (MW732492)	BI	99.9% *A. phagocytophilum* (HM057224)

Organism	Gene (bp)	No. of sequences obtained (GenBank accession No.)	Origin	Percentage identity with published sequence from species (GenBank accession No.)
		1 (MW732493)	IA	99.8% *A. phagocytophilum* (CP046639)
		2 (MW732494)	BI	99.8% *A. phagocytophilum* (AF478553)
		1 (MW732495)	BI	99.9% *A. phagocytophilum* (CP046639-AY281831)
***Ehrlichia* sp.**	*groELS* (1215)	1[4] (MW732496)	BI	99.5% *Ehrlichia* sp. (JX402611)
	gltA (756)	1	BI	100% *Ehrlichia canis* (CP000107)
***Spiroplasma* sp.**	*rpoB* (1359)	1	IA	100% *Spiroplasma* strain Bratislava1 (KP967687)
	16S rRNA (461)	2	BI;IA	100% *Spiroplasma* sp. (KP967685-LC388762-LC388760)
***Wolbachia* spp.**	16S rRNA (970)	17[5,6]	BI	100% *Wolbachia* sp. (AY007547)
		5[4] (MW727242)	BI	99.9% *Wolbachia* sp. (AY007547)
		6 (MW727243)	BI	99.9% *Wolbachia* sp. (GQ275137)
		15[3,7]	BI	99.9% *Wolbachia* sp. (AY007547)
		2[3]	BI	99.8% *Wolbachia* sp. (AY007547)
		6[3]	BI	99.6-99.8% *Wolbachia* sp. (AY007547-CP037426-CP001391-CP042904-CP041215-CP011148)

BI: Brownsea Island; IA: Isle of Arran; [1]This sequence reached 99.5% identity with published *Ixodes inopinatus* sequences with accession numbers MW173342 and GU074596; [2]The same sequence was amplified from DNA extracts of wasp eggs (n=1) and larvae (n=1); [3]Sequences with ambiguous bases; [4]With a query cover of 94%, this sample reached identities of 99.4% with *Ehrlichia minasensis* (JX629806) and 97.5% with *Ehrlichia canis* (CP025749); [5]Identical to the only published sequence of a *Wolbachia* from *I. hookeri* (KU255240) but with query cover of only 59.3%; [6]The same sequence was amplified from the DNA extract of *I. hookeri* eggs (n=1) (MW727241) ; [7]One variant was also amplified from the DNA extract of *I. hookeri* larvae (n=1).
